# pH-Responsive and Biodegradable ZnO-Capped Mesoporous Silica Composite Nanoparticles for Drug Delivery

**DOI:** 10.3390/ma13183950

**Published:** 2020-09-07

**Authors:** Minmin Chen, Jinxia Hu, Cancan Bian, Chenghao Zhu, Chen Chen, Zhijun Guo, Zhimin Zhang, Godfred Amfo Agyekum, Zhuoqi Zhang, Xichuan Cao

**Affiliations:** 1 School of Materials and Physics, China University of Mining and Technology, Xuzhou 221116, China; TB17040040b0@cumt.edu.cn (M.C.); TB18040043B4@cumt.edu.cn (J.H.); 14155026@cumt.edu.cn (C.B.); TS18180001A3TM1@cumt.edu.cn (C.C.); zj.guo@cumt.edu.cn (Z.G.); 14174280@cumt.edu.cn (Z.Z.); 2 School of Chemical Engineering and Technology, China University of Mining and Technology, Xuzhou 221116, China; chenghao.zhu@cumt.edu.cn; 3 Department of Cardiology, Affiliated Hospital of Xuzhou Medical University, Xuzhou 221002, China; 502332117113@stu.xzhmu.edu.cn

**Keywords:** mesoporous silica composite nanoparticles (MSCNs), ZnO QDs, pH-responsive biodegradability, pH-responsive drug delivery

## Abstract

As a drug delivery system (DDS), traditional mesoporous silica nanoparticles (MSNs) suffer from bioaccumulation in vivo and premature drug release in systemic circulation due to low degradation rate and lack of protective gatekeeper. Herein, we developed a safe and intelligent DDS with characteristics of pH-responsive biodegradation and controlled drug release based on mesoporous silica composite nanoparticles (MSCNs) capped with ZnO quantum dots (ZnO QDs). Acidic degradable MSCNs were successfully synthesized by doping Ca^2+^ and PO_4_^3−^ into the MSNs’ framework. The in vitro doxorubicin hydrochloride (DOX) release was inhibited at neutral pH 7.4 but triggered significantly at pH 5.0 due to the dissociation of ZnO caps. The internalization behavior and cytotoxicity of 4T1 cells indicated MSCNs-ZnO could efficiently deliver DOX into the cells with significant antitumor activity. Such a DDS with pH-responsive biodegradation and controlled drug release has promising potential for cancer therapy.

## 1. Introduction

Mesoporous silica nanoparticles (MSNs) are widely employed in biomedical fields such as bioimaging [1], drug delivery [2], cancer diagnosis and therapy [3,4]. With huge specific surface area and pore volume, tailorable pore aperture and easy surface modification by well-known chemistries, MSNs show great potential as drug delivery carrier [5,6,7]. It has been reported that chemotherapeutic therapy for current cancer could lead to severe side effects on normal tissues during the systemic administration of toxic drugs [8]. MSNs with the encapsulated drugs inside can effectively avoid premature leakage as well as significantly improve drugs’ efficacy [9,10,11]. However, it is well-known that pure MSNs have a very slow degradation rate in the physiological environment due to their stable framework. The majority of MSNs would accumulate in the reticuloendothelial system (RES) generating long-term tissue toxicity and restricting their clinical application [12,13]. Thus, MSNs with degradability in vivo could be a promising drug carrier in clinical application.

Organic [14,15] or inorganic entities [16,17] were doped into the MSNs’ framework to transform structural features, which enabled composite nanoparticles more sensitive to the tumor microenvironment (TME), (low pH [18], high reducibility [19], high expressed enzyme [20]), thus achieving TME-responsive biodegradability. For the organic moieties incorporation, the covalent incorporation of stimuli-responsive bonds was introduced into the MSNs’ framework. Representatively, the MSNs’ structure containing disulfide-/diselenide-bridged bonds [21,22] or phenylene-/oxamide-bridged linkage [23,24] disassociated easily in the situation of the reductive agents or enzymes. Similarly, inorganically doped MSNs have been shown to enhance silica degradability. Inorganic components such as zirconium [25], iron [26,27], manganese [28] and calcium oxides [29] doped into MSNs’ framework alter the local Si-O-Si bonds. Interestingly, these inorganic ions could get away from the framework in TME, which results in a partial interior cavity collapse, endowing these doped MSNs with superior biodegradability and better drug release characteristics than traditional pure MSNs. Among these doping inorganic components, as one of the indispensable nutrients in the human body, calcium (Ca) ion has been extensively used as the doping element to improve the dissolution rate of silica without additional toxicity. In particular, the formed Si-O-Ca bond is sensitive to the acidic conditions, thus making the Ca-doped MSNs’ pH-triggered degradation possible. Zhang et al. developed a pH-triggered hybrid drug carrier (MSNs/HAP) by incorporating hydroxyapatite into MSNs using a one-step procedure [30]. It was demonstrated that MSNs/HAP not only exhibited a higher drug loading content but also displayed excellent degradability due to the escape of Ca^2+^ from the network in the acidic environment. Analogously, Xu et al. explored a mesoporous silica-calcium phosphate (MS-CAP) hybrid nanocarrier with a fast pH-responsive biodegradation in 24 h by doping CAP precursors (Ca^2+^ and PO_4_^3−^) into a silica matrix [31]. Although these as-prepared hybrid MSNs have good degradability, the loaded drug molecules in the interior space would encounter the issue of premature leakage through the open pores into systemic circulation.

Thus, it is vitally important to avoid drug leakage, because this not only can enhance the drug efficacy with accumulated drug released at tumor sites but also reduce side effects on normal tissues. Current exploration of significative stimuli-responsive MSN drug delivery systems (DDSs) with nearly “zero premature release” has attracted widespread attention. The vast quantity of reactive silanol groups (Si-OH) on the surface of MSNs gives permission for modification with various designed nanovalves to respond to different types of stimuli involving light, thermal, temperature, redox, enzymes, pH etc. [32,33,34] for the on-demand release. Of these stimuli reported previously, pH stimuli-response was commonly applied due to the lower pH in TME than that in normal tissues, and the even lower pH value in the endosome and lysosome [18,35]. This arouses researchers’ interest to exploit more appropriate and efficient pH-responsive nanovalves. ZnO quantum dots (ZnO QDs) with the merits of straightforward synthesis procedure, low-cost, health-friendly, etc., are capable of plugging drugs to avoid premature release. More importantly, they are stable and safe in a neutral environment and can readily be dissolved in an acidic environment, hence promoting them as the outstanding candidates of pH-responsive gatekeepers [36,37,38].

**Scheme 1:**
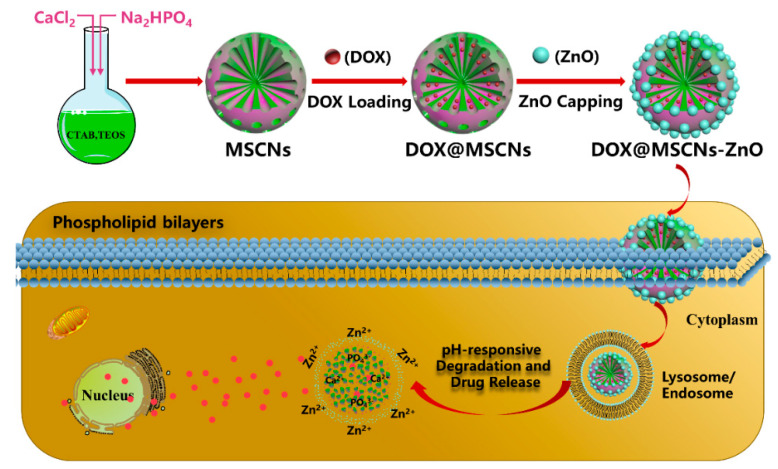
Schematic illustration of ZnO-capped mesoporous silica composite nanoparticles (MSCNs) drug delivery system (DDS) and its procedure of pH-responsive degradation and drug release. DOX: doxorubicin hydrochloride.

In this study, a novel pH-responsive degradable DDS was fabricated based on mesoporous silica composite nanoparticles (MSCNs) bearing ZnO QDs nanovalves. As shown in Scheme 1, the degradable MSCNs were prepared by doping Ca^2+^ and PO_4_^3−^ into MSN framework by a one-step method. The anticancer DOX as a model drug was loaded into the pores of the MSCNs and capped by ZnO QDs nanovalves (DOX@MSCNs-ZnO). Once DOX@MSCNs-ZnO were internalized into 4T1 (mouse breast cancer cells), under the acidic endocytic vesicles, pores were gradually uncapped as ZnO nanovalves dissolved, then the entrapped DOX was released from the pores and effectively inhibited cancer cells’ growth. Simultaneously, the endocytosed MSCNs were disintegrated into small pieces and excreted outside. Such DDSs “MSCNs-ZnO” showed excellent pH-responsive biodegradability and drug-release behavior, and could act as promising carriers to combat a tumor.

## 2. Materials and Methods

### 2.1. Materials

Zinc acetate, calcium chloride anhydrous (CaCl_2_), lithium hydroxide (LiOH) and *N*,*N*-dimethyl formamide (DMF) were purchased from Xilong Chemical Industry (Shantou, China). 3-aminopropyltriethoxysilane (APTES), sodium phosphate dibasic dodecahydrate (Na_2_HPO_4_∙12H_2_O) and hexane were purchased from Aladdin (Shanghai, China). Cetyltrimethylammonium bromide (CTAB) was purchased from Thermo Fisher Scientific (Shanghai, China). Tetraethylorthosilicate (TEOS), ammonia (NH_3_∙H_2_O), absolute ethanol, succinic anhydride and 1-ethyl-3-(3-dimethylaminopropyl) carbodiimide hydrochloride (EDC) were purchased from Sinopharm (Beijing, China).

Doxorubicin hydrochloride (DOX) was purchased from Adamas-beta (Shanghai, China). Dulbecco’s modified eagle medium (DMEM) was purchased from Hyclone (Logan, UT, USA). Fetal bovine serum (FBS) was purchased from Zhejiang Tianhang biological technology (Hu Zhou, China). 2-(4-amidinophenyl)-6-indolecarbamidine dihydrochloride (DAPI) and penicillin-streptomycin solution were purchased from Beyotime (Shanghai, China). A cell counting kit-8 (CCK-8) was purchased from DOJINDO Laboratories (Kumamoto, Japan). The prolong antifade kit was purchased from Life Technologies (Eugene, OR, USA) and 4% paraformaldehyde was purchased from Vicmed (Xuzhou, China).

### 2.2. Synthesis of ZnO QDs

Zinc acetate, 1.32 g, was refluxed in 40 mL ethanol at 78 °C for 3 h under vigorous stirring (denoted as solution A). Lithium hydroxide, 0.3024 g, was dissolved in 20 mL ethanol (denoted as solution B). Solution A was cooled to 20 °C followed by adding solution B with vigorous stirring for 20 min to obtain ZnO QDs [39]. The ZnO QDs solution was then dispersed in hexane till the color turned white. Afterwards, the ZnO QDs were washed with ethanol twice and centrifuged at 10,000 r/min for 5 min followed by vacuum drying. To the anchored amino group with ZnO QDs (ZnO-NH_2_), the as-prepared ZnO QDs were well-ultrasonically dispersed in 20 mL DMF followed by addition of 100 μL APTES, then the mixture was stirred for 20 min at 120 °C. The ZnO-NH_2_ QDs were obtained by washing with DMF twice and centrifuging at 10,000 r/min for 5 min.

### 2.3. Synthesis and Functionalization of MSCNs

#### 2.3.1. Synthesis of MSCNs

MSCNs were prepared by doping CaCl_2_ and Na_2_HPO_4_ during the synthetic process of MSNs based on the Stöber approach [40]. CTAB, 0.20 g, and 0.18 g Na_2_HPO_4_∙12H_2_O were dissolved in 70 mL deionized water followed by adding 20 mL ethanol and 0.27 mL ammonia. The mixture was stirred magnetically for 30 min at 40 °C. Then 1 mL TEOS was dropwise added into the solution with vigorously stirring. Aqueous solution, 1 mL, containing 0.083 g CaCl_2_ was immediately introduced into the solution and stirred for 24 h. The resulted product MSCNs-CTAB were isolated by centrifugation at 8000 r/min for 10 min and washed three times with deionized water and ethanol alternately. To remove surfactant CTAB, the final MSCNs were calcined at 550 °C for 6 h with a heating rate of 1 °C/min.

#### 2.3.2. Amino Functionalization of MSCNs

For the synthesis of amine groups modified MSCNs (MSCNs-NH_2_), 100 mg MSCNs were dispersed in 20 mL ethanol containing 1 mL APTES. The mixture was continually reacted at room temperature by stirring overnight. MSCNs-NH_2_ were collected by centrifuging at 8000 r/min for 10 min and washing with deionized water and ethanol thrice alternately.

#### 2.3.3. Carboxyl Functionalization of MSCNs

The carboxyl group modified-MSCNs (MSCNs-COOH) were synthesized based on the previous report with slight modification [41]. MSCNs-NH_2_ were dispersed in 10 mL DMF including 86 mg succinic anhydride, which was vigorously stirred at room temperature for 24 h under nitrogen protection. Then the as-prepared MSCNs-COOH were washed with deionized water and ethanol thrice respectively.

### 2.4. Biodegradation of MSCNs in PBS Solution and in Cells

MSCNs, 20 mg, were suspended in 20 mL phosphate-buffered saline (PBS) with pH value of 5.0 and 7.4. The mixture was continuously shaken at 37 °C for days. At 1, 3 and 7 d, the nanoparticles in the pH 5.0 and 7.4 were collected for TEM and SEM observation. Simultaneously, the supernatant was collected at fixed times (1 d, 3 d, 5 d, 7 d) to detect the cumulative dissolved rate of Ca^2+^ by inductively coupled plasma-optical emission spectrometer (ICP-OES).

The intracellular biodegradation behavior of the MSCNs was evaluated by bio-TEM observation. Typically, 100 μg/mL MSCNs dispersed in DMEM were co-cultured with 4T1 cells for 1, 3 and 7 d. The cells were harvested by centrifugation at 1000 r/min for 5 min and fixed with 2.5% glutaraldehyde. Then ultrathin cell sections were cut for bio-TEM characterization.

### 2.5. ZnO Capped DOX@MSCNs

#### 2.5.1. Synthesis of DOX@MSCNs

DOX, 20 mg, was dispersed in 40 mL of pH 7.4 PBS followed by adding 80 mg MSCNs-COOH. The mixture was shaken at 25 °C for 24 h. The DOX-loaded MSCNs (DOX@MSCNs) were collected by centrifugation at 8000 r/min for 5 min and dried under vacuum. The absorbance of the supernatant was measured by UV-vis spectrophotometer at 480 nm, the loading amount (mg/g) and efficiency (%) of DOX were calculated by the following Equations (1) and (2):

(1) $$\mathrm{DOX}\;\mathrm{loading}\;\mathrm{amount}\;\left(\mathrm{mg}/g\right)=\frac{\mathrm{Mass}\;\mathrm{of}\;\mathrm{DOX}\;\mathrm{loading}\;\mathrm{into}\;\mathrm{particles}\;(\mathrm{mg})}{\mathrm{Mass}\;\mathrm{of}\;\mathrm{particles}\;(g)}$$

(2) $$\mathrm{DOX}\;\mathrm{loading}\;\mathrm{efficiency}\;\left(\%\right)=\frac{\mathrm{Mass}\;\mathrm{of}\;\mathrm{DOX}\;\mathrm{loading}\;\mathrm{into}\;\mathrm{particles}\;(\mathrm{mg})}{\mathrm{Mass}\;\mathrm{of}\;\mathrm{DOX}\;\mathrm{loaded}\;\mathrm{particles}\;(\mathrm{mg})}\times 100\%$$

#### 2.5.2. ZnO-Capped DOX@MSCNs

The above DOX@MSCNS were dispersed in 10 mL aqueous solution containing 50 mg ZnO-NH_2_ QDs and 60 mg EDC. After stirring at room temperature for 5 h, the precipitate was centrifuged (5000 r/min, 5 min) and washed several times with deionized water until the supernatant became transparent. The ZnO-capped drug loaded particles (DOX@MSCNs-ZnO) were collected after freeze-drying. The ZnO-capped MSCNs (MSCNs-ZnO) were prepared using the same synthetic approach as DOX@MSCNs-ZnO.

### 2.6. In Vitro pH-Responsive Drug Release of DOX@MSCNs-ZnO

To investigate the release behavior of DOX from DOX@MSCNs-ZnO, 4 mg of DOX@MSCNs-ZnO were dispersed into 4 mL pH 5.0 and 7.4 PBS, respectively and placed in a 37 °C constant temperature shaker to release. The supernatant was gathered at 1 h, 4 h, 8 h, 12 h, 24 h, 36 h and 48 h, respectively by centrifugation at 8000 r/min for 5 min. Subsequently, the supernatant was supplemented to 4 mL for releasing again.

As control, the release behavior of DOX from DOX@MSCNs (without ZnO capping) was also performed with the same experimental process as that of DOX@MSCNs-ZnO. The cumulative release rate (%) of DOX was calculated by the absorbance at 480 nm of the supernatant according to the following Equation (3).

(3) $$\mathrm{DOX}\;\mathrm{cumulative}\;\mathrm{release}\;\mathrm{rate}\;\left(\%\right)=\frac{\mathrm{Mass}\;\mathrm{of}\;\mathrm{DOX}\;\mathrm{released}\;\mathrm{from}\;\mathrm{particles}\left(\mathrm{mg}\right)}{\mathrm{Mass}\;\mathrm{of}\;\mathrm{DOX}\;\mathrm{loaded}\;\mathrm{into}\;\mathrm{particles}\;(\mathrm{mg})}\times 100\%$$

### 2.7. Cell Culture

4T1 cells (mouse breast cancer cell line) were kindly presented by Jiangsu Center for the collaboration and innovation of cancer biotherapy from the cell bank of the Chinese Academy of Sciences (Shanghai, China), which were incubated with DMEM containing 10% FBS and 1% penicillin-streptomycin under a humidified 5% CO_2_ atmosphere at 37 °C.

### 2.8. Intracellular Endocytosis of DOX@MSCNs-ZnO

The 4T1 cells were seeded into a 24-well plate at the concentration of 1 × 10^4^ per well, and were treated with 100 μg/mL of DOX@MSCNs-ZnO in DMEM. At different intervals (0.5, 1, 4 and 8 h), after discarding the medium, the cells were washed with pH 7.4 PBS three times and then fixed with 4% (g/mL) paraformaldehyde for 30 min. Subsequently, after removal of paraformaldehyde and washing with pH 7.4 PBS, the nuclei were stained by DAPI (blue) for 10 min. With the cells sealed with a prolong antifade kit, cellular uptake behavior was observed by confocal laser scanning microscopy (CLSM).

### 2.9. In Vitro Cytotoxicity Assays

In vitro cell viability of the 4T1 cells was measured by CCK-8 assay. The cells were seeded into a 96-well plate with 3.0 × 10^4^ cells per well and incubated overnight at 37 °C. The samples were divided into two groups involving the first group of ZnO and Zn^2+^ (ZnCl_2_) with concentrations 0.1, 0.5, 2.5, 5, 25, 50 and 100 μg/mL, and the second group of MSCNs, MSCNs-ZnO, DOX@MSCNs-ZnO, DOX@MSCNs with concentrations 1, 5, 10, 25, 50, 100 and 200 μg/mL and free DOX with the corresponding concentrations: 0.188, 0.94, 1.88, 4.7, 9.4, 18.8 and 37.6 μg/mL. The 4T1 cells were treated with the above samples for 24 h. Afterwards, 10 μL CCK-8 was added into the medium per well followed by incubation for another 2 h. The optical density (OD) of the samples was measured using a microplate spectrophotometer at 450 nm. The cell viability was calculated based on the following Equation (4):

(4) $$\mathrm{Cell}\;\mathrm{viability}\;\left(\%\right)=\;\frac{\mathrm{ODe}-\mathrm{ODb}}{\mathrm{ODc}-\mathrm{ODb}}\times 100\%$$

where ODe is the absorbance value of experimental well (DMEM with cells, CCK-8 and samples), ODc is the absorbance value of control well (DMEM with cells, CCK-8, and without samples) and ODb is the absorbance value of blank well (DMEM without cells and CCK-8). Data statistics were calculated based on six independent results.

### 2.10. Characterization of Nanoparticles

Scanning electron microscope (SEM) images were taken with SU8220 microscope (Hitachi, Ibaraki, Japan) at an accelerating voltage of 10 Kv. SEM elements mapping images were obtained on an energy dispersive spectrometer (EDS) with XFlash Detector 6|30 (Bruker Nano GmbH, Karlsruhe, Germany). Transmission electron microscopy (TEM) was carried out at Tecnai G2 F20 microscope (FEI, Hillsboro, OR, USA) at an exciting voltage of 200 kV. The powder X-ray diffraction (XRD) patterns were recorded on a D8 ADVANCE (Bruker, Karlsruhe, Germany) X-ray diffractometer with Cu-Kα radiation (λ = 1.5418 Å) at the scanning step of 0.02°. The specific surface area, pore volume and pore size of the particles were measured at ASAP 2020 instrument (Micromeritics, Norcross, GA, USA), which was calculated applying the Brunauer-Emmett-Teller (BET) and the Barrett-Joyner-Halenda (BJH) method. The hydrodynamic diameter of the particles was detected by NanoBrook Omni instruments (Brookhaven Instruments Corporation, Holtsville, NY, USA). The element component of nanoparticles was carried out on ESCALAB250Xi X-ray photoelectron spectroscopy (XPS). The drug absorbance was examined by Evolution 60 ultraviolet-visible (UV-vis) spectrophotometer (Thermo, Walsham, MA, USA). An inductively coupled plasma-optical emission spectrometer (ICP-OES, Optima8000, PerkinElmer, Waltham, MA, USA) was used to analyze the amount of dissolved ion. The confocal laser scanning microscopy (CLSM) was carried out on ZEISS LSM880 (Jena, Germany) to observe the intracellular endocytosis of the drug-loaded particles with a blue fluorescence excitation wavelength of 405 nm and a red fluorescence excitation wavelength of 561 nm. The cell sections were observed by a JEM1010 bio-TEM (JEOL, Tokyo, Japan) to evaluate the intracellular biodegradation of nanoparticles.

### 2.11. Statistical Analysis

Data were statistically analyzed by OriginPro (version 8.0, OriginLab, Northampton, MA, USA) and expressed as mean ± standard deviation (SD) at least three times.

## 3. Results and Discussions

### 3.1. Synthesis and Characterization of ZnO QDs

#### 3.1.1. Morphology and Particle Size of ZnO QDs

**Figure 1:**
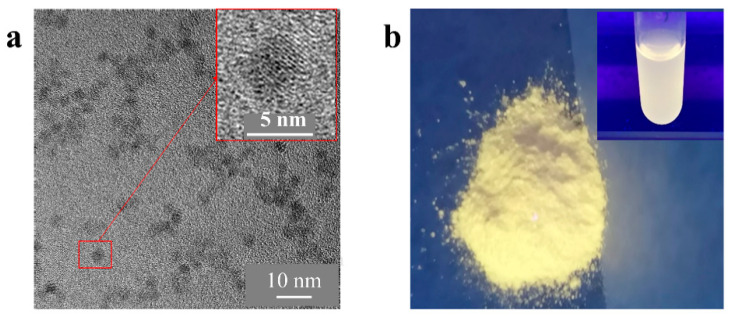
(**a**) TEM images of ZnO QDs. (**b**) Digital photographs for power and aqueous solution (the inset image) of ZnO quantum dots (QDs) under 365 nm UV irradiation.

The morphology and particle size were observed by TEM. It can be seen from Figure 1a that ZnO QDs were approximately spherical in shape and had an average particle size of 4 nm with good dispersion. The powder and aqueous solution of the ZnO QDs exhibited yellow-green fluorescence under UV light at 365 nm (Figure 1b).

#### 3.1.2. Elemental and Structural Analysis of ZnO QDs

**Figure 2:**
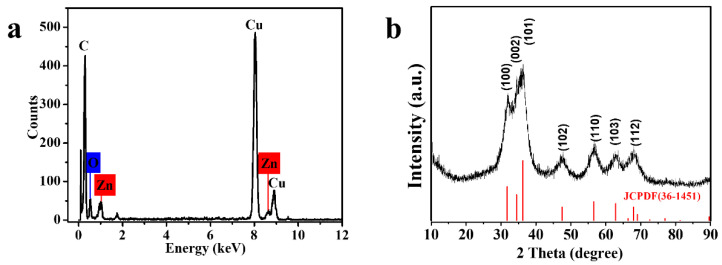
(**a**) EDS and (**b**) wide-angle x-ray diffraction (WAXRD) of ZnO QDs.

Elemental composition of ZnO QDs was analyzed by EDS. As shown in Figure 2a, copper (Cu), carbon (C), zinc (Zn) and oxygen (O) elements were detected. Cu and C elements were mainly from the network material Cu mesh and carbon film while the Zn and O elements were derived from ZnO QDs. The wide-angle x-ray diffraction (WAXRD) of ZnO QDs is shown in Figure 2b, which could match well with the standard card Joint Committee on Powder Diffraction Standards (JCPDS) 36-1451 of the hexagonal wurtzite structure.

#### 3.1.3. Acidic Degradation Behavior of ZnO QDs

**Figure 3:**
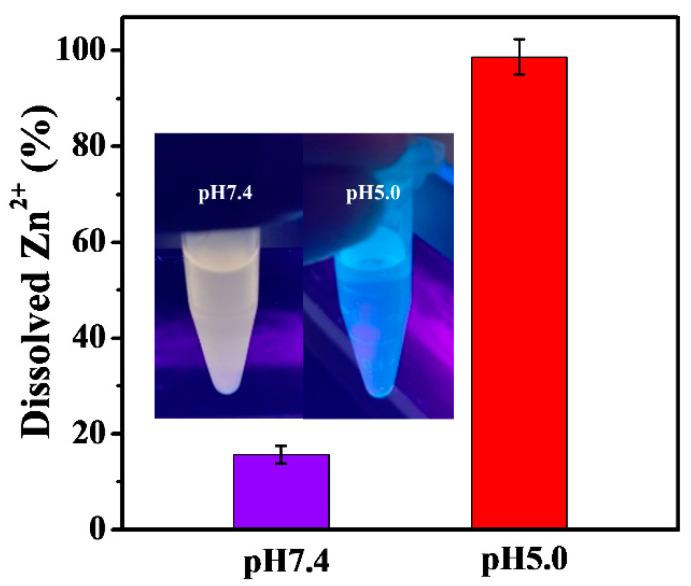
The percentage of dissolved Zn^2+^ in pH 7.4 and pH 5.0 phosphate-buffered saline (PBS) and the digital photographs (the inset images) of ZnO QDs dispersed in pH 7.4 and pH 5.0 PBS under 365 nm UV irradiation.

As shown in Figure 3, the percentage of dissolved Zn^2+^ was detected by ICP-OES after dispersing ZnO QDs in PBS at pH 5.0 for 30 min and pH 7.4 for 7 d. It was calculated that 98.7% ± 3.6 of Zn^2+^ was found in pH 5.0 PBS after 30 min immersion, which was ascribed to the rapid decomposition of the ZnO QDs in the acidic environment. On the contrary, when immersed in pH 7.4 PBS for a relatively long time (7 d), only 15.7% ± 1.8 of Zn^2+^ dissolved out, indicating ZnO QDs were more stable in the neutral environment. This was further verified by the visualized yellow-green fluorescence in pH 7.4 PBS and no fluorescence in pH 5.0 PBS under 365 nm UV irradiation (as shown in the inset images in Figure 3). These acidic-triggered, decomposed ZnO QDs could be developed as the pH-responsive gate to seal a drug in the pores of MSCNs and release the entrapped drug in the acidic compartment of the cancer cells.

### 3.2. Synthesis and Characterization of MSCNs

#### 3.2.1. Morphology and Particle Size of MSCNs

**Figure 4:**
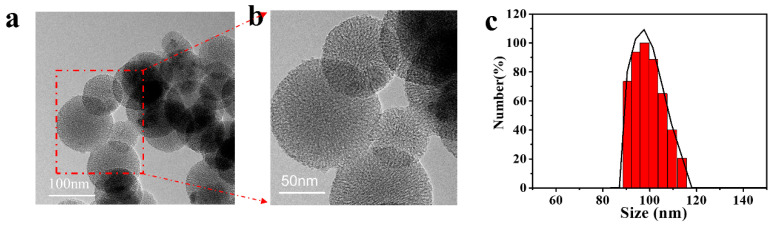
(**a**,**b**) TEM images, (**c**) size distribution of the MSCNs.

The degradable MSCNs were prepared by adding CaCl_2_ and Na_2_HPO_4_ during the synthesis process of MSNs. The doping of Ca^2+^ and PO_4_^3−^ into the framework of MSNs was expected to transform the original stable structure and make it possible to decompose MSCNs in an acidic environment. As shown in TEM images (Figure 4a,b), the as-prepared MSCNs were spherical in shape and about 100 nm in average diameter with good dispersion and well-ordered porous structure. The average size distribution with diameter of 100 nm was confirmed by dynamic light scattering (DLS) technique (Figure 4c).

#### 3.2.2. Structure, Specific Surface Area and Pore Diameter of MSCNs

**Figure 5:**
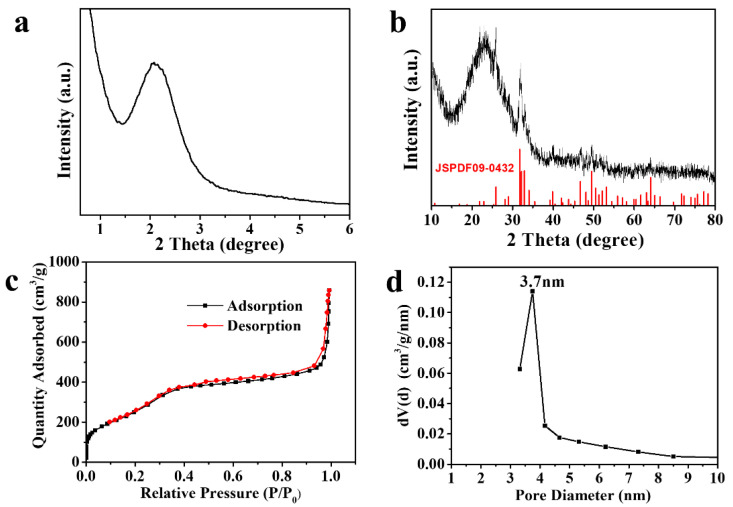
(**a**) Small-angle XRD (SAXRD) pattern, (**b**) WAXRD pattern, (**c**) nitrogen adsorption desorption isotherms, (**d**) pore size distribution diagrams of MSCNs.

Figure 5a revealed the small-angle XRD (SAXRD) of the MSCNs. The emergence of the peak at 2θ of 2.2° was corresponding with the (100) crystal plane of mesoporous material indicating the uniform mesostructure of MSCNs as pure MSNs. The addition of inorganic ion would not affect the architecture. In Figure 5b WAXRD pattern, the 2θ of 22° was the amorphous characteristic peak of silica. The other occurring peaks could be attributed to hydroxyapatite consisting of Ca^2+^ and PO_4_^3−^, which was in agreement with the standard card JCPDS 09-0432 of hexagonal hydroxyapatite, indicating the successful incorporation of Ca_3_(PO_4_)_2_ into the pore walls of the MSNs. Under an acidic environment, Ca_3_(PO_4_)_2_ was prone to be hydrolyzed into Ca^2+^ and PO_4_^3−^, which would lay the foundation for MSCNs to achieve acidic degradation. The nitrogen adsorption desorption curve of MSCNs (Figure 5c) was a type IV curve, further implying the synthesized MSCNs have a mesoporous structure. The calculated BET specific surface area of the MSCNs was 973.3 m^2^/g, and the pore volume was 0.83 cm^3^/g with the mean pore diameter of 3.7 nm (Figure 5d). MSCNs doped with Ca^2+^ and PO_4_^3−^ still maintained the excellent features (ordered mesoporous structure, high specific surface area and pore volume, suitable pore diameter) and had high drug-loading capacities as pure MSNs.

#### 3.2.3. Elemental Analysis of MSCNs

**Figure 6:**
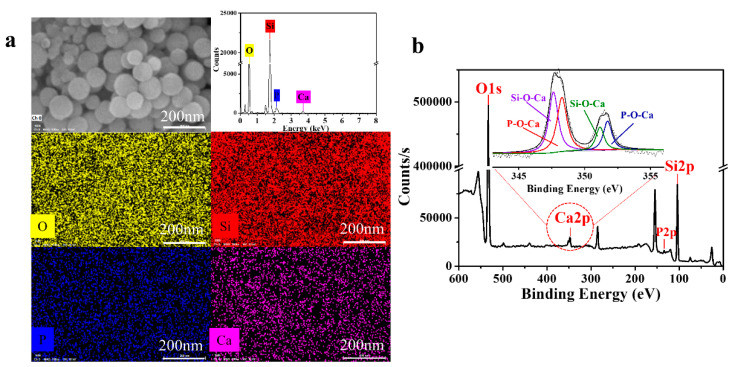
(**a**) Element mappings of O, Si, P and Ca in MSCNs and EDS results of the MSCNs. (**b**) XPS spectra of MSCNs (inset image: high-resolution XPS spectrum of Ca 2p region of the MSCNs).

SEM elemental mapping and an EDS curve were applied to investigate the elemental distribution of the MSCNs. Calcium (Ca) and phosphorus (P) signals were detected as uniformly distributed in MSCNs, indicating the successful incorporation (Figure 6a). The elemental components and chemical structure of the MSCNs were further investigated by XPS (Figure 6b). Figure 6b clearly presents the significant peaks at 104, 139, 354 and 538 eV corresponding to Si 2p, P 2p, Ca 2p and O 1s respectively. A close-up view of the Ca 2p peak is divided into four characteristic peaks at 351.7, 351.2, 348.3 and 347.6 eV attributing to the Si-O-Ca and P-O-Ca bond in the MSCNs’ framework. Both results demonstrated Ca and P were successfully incorporated into the silica network and replaced some Si atoms in Si-O-Si group. It was reported that the condensation degree of silica network strongly affected its degradability, thus the low density of Si-O-Si could promote the degradable rate [42,43]. Ca and P in the network decreased the cross-linking degree of Si-O-Si. In addition, both doped-Si-O-Ca and -P-O-Ca are pH-sensitive and easily hydrolyzed to Ca^2+^ and PO_4_^3−^ under an acidic environment. Thus, to a greater extent, the dual-combination action accelerated the MSCNs’ degradation.

### 3.3. The Biodegradation Behavior of MSCNs

#### 3.3.1. Degradation of MSCNs in PBS Solution

**Figure 7:**
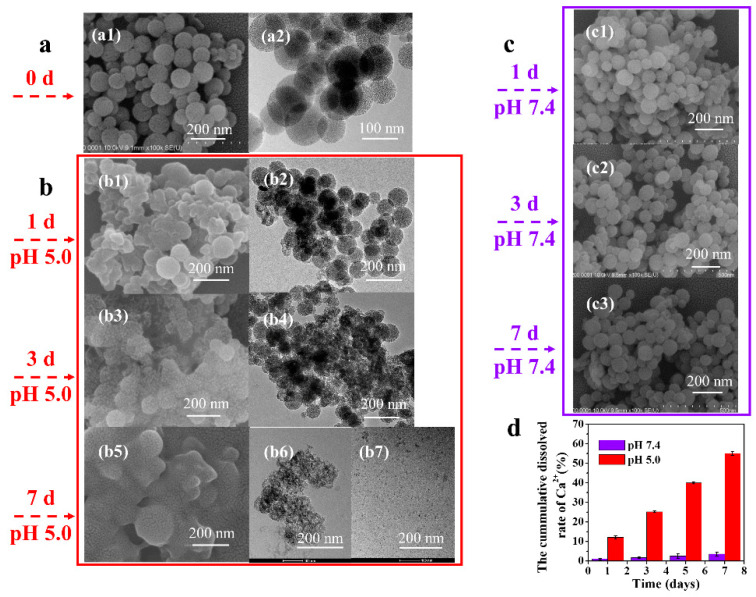
SEM (**a1**, **b1**, **b3**, **b5**, **c1**, **c2**, **c3**) and TEM (**a2**, **b2**, **b4**, **b6**, **b7**) images of MSCNs immersed in PBS at pH 5.0 and 7.4 for varied durations (**a**) 0 d, (**b**) 1, 3 and 7 d in pH 5.0, (**c**) 1, 3 and 7 d in pH 7.4. (**d**) The cumulative dissolved rate of Ca^2+^ in pH 5.0 and 7.4 PBS for 1, 3, 5 and 7 d.

Since the as-prepared MSCNs were expected to be sensitive to the acidic environment, the in vitro degradation behavior was evaluated in PBS solution at different pH values (5.0 and 7.4). MSCNs were immersed in pH 5.0 and 7.4 PBS for 1, 3 and 7 d. The changes of the particles’ morphology were tracked by SEM and TEM images. The MSCNs were spherical with a smooth surface and highly ordered mesoporous structure before degradation (Figure 7a). In pH 5.0 PBS solution, the MSCNs underwent significant time-dependent degradation (Figure 7b). After 1 d immersion (Figure 7b1,b2), there was partial structural breakdown of some nanoparticles with morphology changing from spherical to blurred. With time, more considerably biodegraded nanoparticles were found, which exhibited severe structural damages after 3 d (Figure 7b3,b4). When immersion time extended to 7 d, the spherical morphology of the particles was completely invisible (Figure 7b5) and also no mesoporous structure was observed (Figure 7b6), indicating that a complete collapse had occurred. Notably, some of them even disintegrated into smaller fragments as shown in Figure 7b7, which provided a prerequisite for metabolizing particles out of the body in a relatively short time. In contrast, no evident morphological changes were found in pH 7.4 PBS solution (Figure 7c). Though after 7 d immersion, MSCNs still maintained the spherical morphology and intact structure, indicating that the particles were stable in the neutral environment. Moreover, the acid-sensitivity characteristic of the MSCNs was further corroborated by the Ca^2+^ dissolution amount from the framework in pH 5.0 and 7.4 PBS by ICP-OES detection (Figure 7d). The final cumulative dissolved rate of Ca^2+^ was calculated to be 55% ± 1.0 in pH 5.0 and less than 5% ± 0.9 Ca^2+^ in pH 7.4, which further demonstrated the obvious acid-sensitivity degradation characteristic of MSCNs.

#### 3.3.2. Degradation of MSCNs in 4T1 Cells

**Figure 8:**
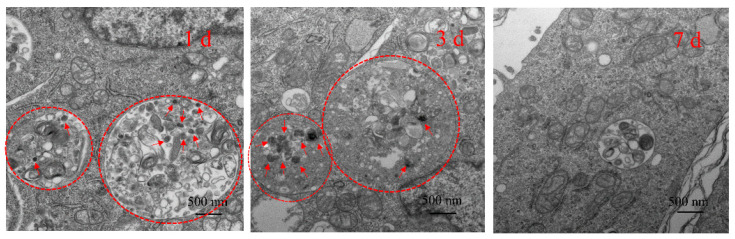
Bio-TEM images of intracellular biodegradation for MSCNs culturing with 4T1 cells for 1, 3 and 7 d.

Furthermore, the biodegradation behavior of MSCNs intracellularly was observed by Bio-TEM after co-cultivation with 4T1 cells for 1, 3 and 7 d. As shown in Figure 8, the MSCNs could be efficiently internalized into cells and the evident time-dependent biodegradation was found in the intracellular microenvironment, which was consistent with the above in vitro degradation at pH 5.0. After 1 d incubation, the endocytosed nanoparticles (shown by red arrow) were evenly dispersed in cells and kept their intact structure with a spherical shape and clear edge. When co-cultivation time extended to 3 d, the spherical morphology of MSCNs became blurred with structural collapse indicating the endocytosed MSCNs gradually underwent degradation in the acidic compartments. After 7 d co-cultivation, it was difficult to find nondegraded particles inside the cells, suggesting MSCNs were almost completely degraded and excreted out of the cells. The endocytosed MSCNs experienced this significant degradation behavior at a cellular level. As known, the pH value in the endosome or lysosome was low (pH 5.0), which would be the useful factor to prompt the pH-sensitive MSCNs’ biodegradation [34] in cells.

### 3.4. ZnO-Capped DOX@MSCNs

**Figure 9:**
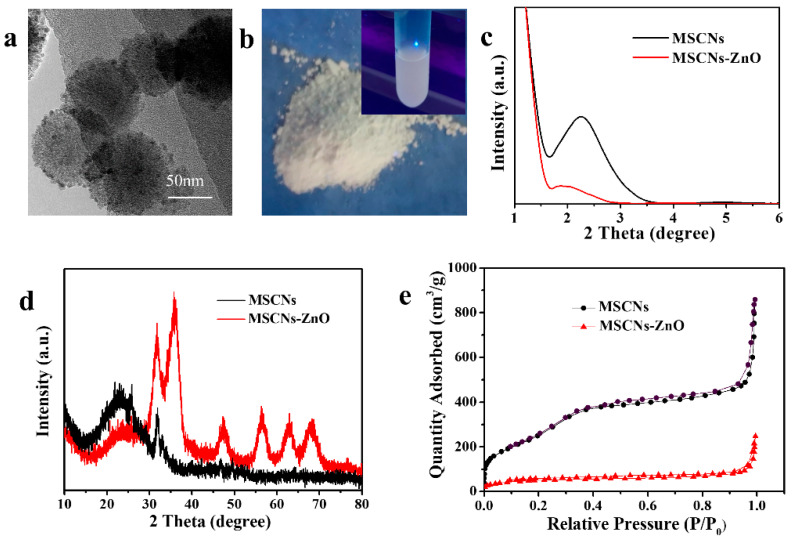
(**a**) TEM images of MSCNs-ZnO. (**b**) Photographs for power and aqueous solution (the inset image) of MSCNs-ZnO under 365 nm UV irradiation. (**c**) SAXRD patterns of MSCNs and MSCNs-ZnO. (**d**) WAXRD patterns of MSCNs and MSCNs-ZnO. (**e**) Nitrogen adsorption desorption isotherms of MSCNs and MSCNs-ZnO.

To prevent drug from premature release, the as-prepared pH-sensitive ZnO QDs were used to block MSCNs pores. The amino-modified ZnO QDs were tethered to the outlet of the MSCNs-COOH nanopores by amidation coupling reaction. The morphology of the MSCNs-ZnO was observed by TEM images (Figure 9a), which evidently displayed that ZnO QDs were distributed on the surface of the MSCNs, realizing the purpose of plugging nanopores. Interestingly, after capping, MSCNs-ZnO exhibited the same yellow-green fluorescence behavior as the pure ZnO QDs, which further verified the presence of ZnO QDs on the surface of the nanoparticles (Figure 9b). Figure 9c displays the SAXRD of MSCNs and MSCNs-ZnO. Since ZnO QDs blocked the pores of MSCNs, the diffraction peak intensity of MSCNs-ZnO was lower than that of the bare MSCNs. WAXRD pattern of MSCNs-ZnO showed the peak overlay of ZnO and MSCNs, attributing to the successfully modified ZnO on the surface of MSCNs (Figure 9d). After the pores were capped with ZnO, the BET-specific area of MSCNs-ZnO reduced to 185 m^2^/g from 973.3 m^2^/g of MSCNs (Figure 9e). Together, a series of significant changes between bare MSCNs and MSCNs-ZnO demonstrated the successful capping of ZnO on MSCNs.

### 3.5. In Vitro pH-Responsive Drug Release of DOX@MSCNs-ZnO

**Figure 10:**
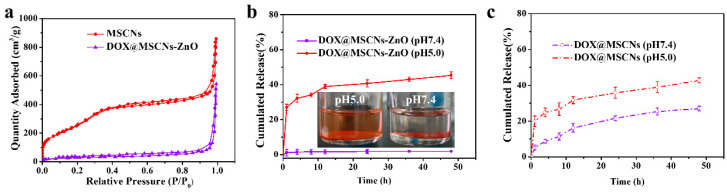
(**a**) Nitrogen adsorption desorption isotherms of MSCNs and DOX@MSCNs-ZnO. (**b**) Cumulative release curve of DOX@MSCNs-ZnO at pH 7.4 and pH 5.0, inset images: photographs of the DOX@MSCNs-ZnO released at pH 7.4 and pH 5.0 for 1 h. (**c**) Cumulative release curve of DOX@MSCNs (without ZnO nanovalves) at pH 7.4 and pH 5.0.

To verify the pH-responsive drug release of MSCNs-ZnO, DOX as the anticancer model drug was loaded into the pores of MSCNs-COOH and subsequently plugged with ZnO QDs. The loading amount and efficiency of DOX were calculated as 231.3 mg/g and 18.8%, respectively. Since DOX was loaded into the interior of pores and the “nanovalves” ZnO sealed the outlet of pores, the BET-specific surface area of DOX@MSCNs-ZnO reduced to 104.4 m^2^/g compared to bare MSCNs (973.3 m^2^/g) (Figure 10a).

To validate the effect of pH-responsive uncapping on drug release, the release behavior of DOX@MSCNs-ZnO was studied in acidic and neutral environments (pH 5.0 and 7.4 PBS solution) as shown in Figure 10b. It can be seen that negligible DOX was released from MSCNs-ZnO in pH 7.4 due to the capped pores with ZnO. However, in the pH 5.0 environment, an initial burst release was found with a cumulative release rate of 27% ± 1.7 at 1 h, which was attributed to the rapid dissolution of ZnO QDs. Furthermore, release tended to reach a saturation at 12 h and the final cumulative release reached 45% ± 2.0 at 48 h. This result was also confirmed by the color change of DOX@MSCNs-ZnO soaked in pH 7.4 and 5.0 for 1 h. As shown in the inset images of Figure 10b, after immersion in pH 7.4, the supernatant remained unchanged, indicating a negligible release of DOX. On the contrary, when immersed in pH 5.0, the solution was gradually transformed from the supernatant liquid to red, suggesting the continuous release of DOX in acidic environment. The lesser released amount in pH 7.4 but significantly increased release in pH 5.0 indicated the acid-responsive release capacity of the ZnO nanovalves.

Figure 10c presents the cumulative release of DOX from DOX@MSCNs without capping with ZnO under the same situation. Apparently, without ZnO nanovalves, the final cumulative release of DOX from DOX@MSCNs reached 27% ± 1.4 in pH 7.4, which was higher than that from DOX@MSCNs-ZnO. It revealed that the ZnO nanovalves could plug drugs into the pores in pH 7.4, preventing the premature drug release into systemic circulation in neutral conditions. The final cumulative drug release amount in pH 5.0 was nearly the same as DOX@MSCNs-ZnO, suggesting that the existence of ZnO nanovalves would not affect the drug release. Thus, considering the above results, the ZnO QDs were excellent candidates as the pH-responsive “on-off” for the drug controlled release system.

### 3.6. Endocytosis of DOX@MSCNs-ZnO

**Figure 11:**
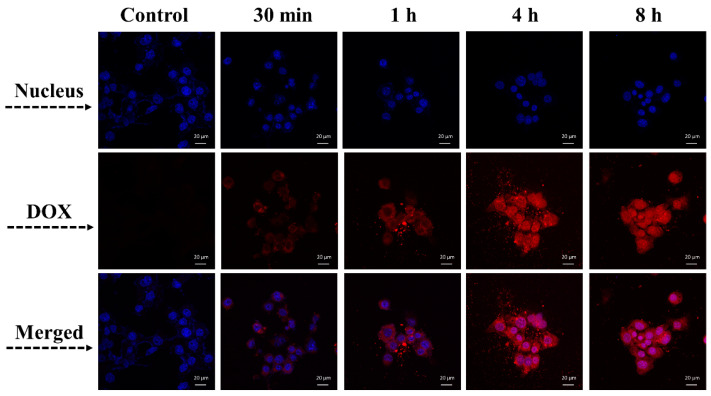
CLSM of DOX@MSCNs-ZnO cultured with 4T1 cells for 30 min, 1 h, 4 h and 8 h. Blue: cell nucleus staining by 2-(4-amidinophenyl)-6-indolecarbamidine dihydrochloride (DAPI). Red: DOX.

The cellular uptake behavior of DOX@MSCNs-ZnO was observed by CLSM after culturing with 4T1 cells for 30 min, 1 h, 4 h and 8 h. As shown in Figure 11, DOX was released over time as MSCNs-ZnO was gradually degraded in the acidic endocytic vesicles. After 30 min and 1 h, part of DOX had begun to be released into the cytoplasm, which was attributed to uncapped pores caused by the rapid dissolution of ZnO. After continuous incubation for 4 h and 8 h, more-and-more DOX was released and distributed evenly throughout the cells. When internalized into endosome or lysosome with lower pH value [34], ZnO nanovalves were rapidly decomposed and the DOX was released from the nanopores intracellularly. Meanwhile, as depicted in the intracellular biodegradation experiment, MSCNs could be degraded into fragments in cells, which further enhanced the drug release. The “DOX@MSCNs-ZnO” DDS could efficiently transport drugs into cancer cells and release drugs into cells in a sustained and pH-stimulated controlled way.

### 3.7. In Vitro Cell Cytotoxicity

**Figure 12:**
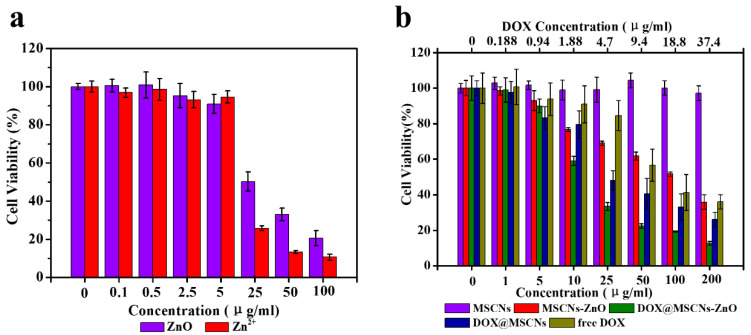
In vitro cell viability of 4T1 cells treated with (**a**) ZnO QDs and Zn^2+^, (**b**) MSCNs, MSCNs-ZnO, DOX@MSCNs-ZnO, DOX@MSCNs and free DOX.

CCK-8 assay was employed to investigate the cytotoxicity of different samples cultured with 4T1 cells for 24 h. Firstly, the cell viability of ZnO and Zn^2+^ (as ZnCl_2_) with different concentrations is shown in Figure 12a. It is clearly seen that both ZnO and Zn^2+^ exhibited similar concentration-dependent tendency. At the concentration of less than 25 μg/mL, both had almost no toxic effect on 4T1 cells. Interestingly, when the concentration surpassed 25 μg/mL, the cell viability declined sharply to 50% ± 5.0 for ZnO and 26% ± 1.2 for Zn^2+^. ZnO QDs dissolved into Zn^2+^ in the acidic environment of cellular compartments. According to previous reports [44,45,46], the accumulation of Zn^2+^ inside cells induced the production of reactive oxygen species (ROS) followed by peroxidation of lipid and damage of DNA. As a result, ZnO QDs had a therapeutic effect on cancer cells.

Figure 12b shows the viability of 4T1 cells in the presence of MSCNs, DOX@MSCNs-ZnO, DOX@MSCNs and free DOX with different concentration. It is clearly seen that as the drug carrier itself, the bare MSCNs had no obvious cytotoxicity even at the concentration of 200 μg/mL, suggesting that MSCNs have excellent biocompatibility and can act as the safe drug vehicle. MSCNs-ZnO exhibited concentration-dependent cytotoxicity, which was mainly caused by ZnO attached on MSCNs. Free DOX, DOX@MSCNs and DOX@MSCNs-ZnO all exhibited significant toxicity with the increased concentration. However, among these, the half-maximal inhibitory concentration (IC_50_) of DOX@MSCNs-ZnO was calculated to be 4.3 μg/mL of equivalent DOX concentration, less than that of DOX@MSCNs (7.3 μg/mL) and free DOX (17.6 μg/mL). At the same DOX concentration, DOX@MSCNs-ZnO can effectively inhibit more cancer cell growth than DOX@MSCNs and free DOX, which was the result of the synergistic anticancer therapeutic effect of the released DOX and ZnO from DOX@MSCNs-ZnO.

## 4. Conclusions

In summary, we developed a safe and intelligent DDS “DOX@MSCNs-ZnO” based on “MSCNs” capping with “ZnO QDs” nanovalves, which possessed the traits of pH-responsive biodegradability and pH-responsive drug release, aiming at overcoming the crucial drawback of bioaccumulation of traditional MSNs and developing the on-demand drug release mechanism. Both ZnO QDs and MSCNs exhibited improved biodegradability in the acidic environment, endowing the DDS with pH-responsive degradation behavior in the acidic TME. Furthermore, the acid degradable ZnO QDs successfully prevented DOX in the pores of MSCNs from premature leakage in neutral environment but effectively triggered the release of DOX in an acidic environment. Besides, MSCNs-ZnO could efficiently transport DOX into cancer cells and showed enhanced cancer cells inhibition. Thus, this DDS with the excellent features of pH-responsive biodegradability, pH controlled drug release, avoiding premature drug leakage and enhancing therapeutic efficiency is expected to have a promising potential in clinical application for cancer therapy.
